# Gene duplication and dosage effects during the early emergence of C_4_ photosynthesis in the grass genus *Alloteropsis*

**DOI:** 10.1093/jxb/ery029

**Published:** 2018-02-01

**Authors:** Matheus E Bianconi, Luke T Dunning, Jose J Moreno-Villena, Colin P Osborne, Pascal-Antoine Christin

**Affiliations:** Department of Animal and Plant Sciences, University of Sheffield, Sheffield, UK

**Keywords:** Biochemical pathway, C_4_ photosynthesis, copy number variation, dosage effect, gene duplication, grasses, low-coverage sequencing

## Abstract

The importance of gene duplication for evolutionary diversification has been mainly discussed in terms of genetic redundancy allowing neofunctionalization. In the case of C_4_ photosynthesis, which evolved via the co-option of multiple enzymes to boost carbon fixation in tropical conditions, the importance of genetic redundancy has not been consistently supported by genomic studies. Here, we test for a different role for gene duplication in the early evolution of C_4_ photosynthesis, via dosage effects creating rapid step changes in expression levels. Using genome-wide data for accessions of the grass genus *Alloteropsis* that recently diversified into different photosynthetic types, we estimate gene copy numbers and demonstrate that recurrent duplications in two important families of C_4_ genes coincided with increases in transcript abundance along the phylogeny, in some cases via a pure dosage effect. While increased gene copy number during the initial emergence of C_4_ photosynthesis probably offered a rapid route to enhanced expression, we also find losses of duplicates following the acquisition of genes encoding better-suited isoforms. The dosage effect of gene duplication might therefore act as a transient process during the evolution of a C_4_ biochemistry, rendered obsolete by the fixation of regulatory mutations increasing expression levels.

## Teaser

Recurrent duplication of two key C_4_ genes during the emergence of a C_4_ cycle in the grass genus *Alloteropsis* is correlated with increases in transcript abundance

## Introduction

C_4_ photosynthesis is a complex trait that results from the co-ordinated action of multiple biochemical and anatomical components to concentrate CO_2_ at the site of Rubisco, increasing photosynthetic efficiency under warm and dry conditions (Hatch, 1987; Sage, 2004). Despite its complexity, the C_4_ trait evolved multiple times independently in several groups of angiosperms (Sage *et al.*, 2011). All enzymes required for the C_4_ pathway were present in non-C_4_ ancestors, where they were responsible for different, non-photosynthetic functions (Sage, 2004; Aubry *et al.*, 2011). The evolution of C_4_ photosynthesis consequently required the co-option of these enzymes into new functions, followed by changes in their expression patterns and/or catalytic properties (Bläsing *et al.*, 2000; Tausta *et al.*, 2002; Gowik *et al.*, 2004; Akyildiz *et al.*, 2007; Christin *et al.*, 2007; Hibberd and Covshoff, 2010; Huang *et al.*, 2017). It has been hypothesized that this massive co-option was facilitated by gene duplication, with one of the duplicates acquiring the novel C_4_ function via neofunctionalization while the other continued to fulfil the ancestral function (Monson, 1999, 2003; Sage, 2004). However, recent genomic studies have not supported this hypothesis of genetic redundancy facilitating neofunctionalization, meaning that the genomic mechanisms enabling the acquisition of novel functions during C_4_ evolution remain largely unknown.

Most C_4_-related enzymes are encoded by multigene families, with numerous paralogues that emerged via multiple rounds of whole-genome and single-gene duplications during angiosperm diversification (Wang *et al.*, 2009; Christin *et al.*, 2013, 2015; Huang *et al.*, 2017). However, the number of paralogues within each of these gene families does not differ significantly between C_3_ and C_4_ species (Williams *et al.*, 2012; van den Bergh *et al.*, 2014). Comparative genomics on a handful of grasses have identified duplicates that have been retained on branches leading to two C_4_ origins, but these did not encode enzymes necessarily involved in the C_4_ cycle (Emms *et al.*, 2016). Indeed, investigations focusing on genes families with a known function in C_4_ photosynthesis indicate that the gain of a C_4_-specific function was generally not directly preceded by a gene duplication event (Christin *et al.*, 2007, 2009; Wang *et al.*, 2009). Although the creation of a large reservoir of ancient duplications might still be important (Monson, 2003), these various lines of evidence suggest that C_4_ evolution did not consistently involve duplication followed by neofunctionalization of one copy while the other retained the ancestral function. However, gene duplication might still have played a role in the initial emergence of C_4_ photosynthesis, via a combination of dosage effects and neofunctionalization.

Small-scale or whole-genome duplications are generally expected to increase transcript abundance through a gene dosage effect (Otto *et al.*, 1986; Kondrashov *et al.*, 2002; Conant and Wolfe, 2008; Conant *et al.*, 2014). Instances of retention of duplicated genes due to a dosage effect on expression levels have been reported for a number of adaptive traits, which include insecticide resistance in the *Culex* mosquito (Mouchès *et al.*, 1986), cold protection in Antarctic fishes (Chen *et al.*, 2008), and nematode resistance in soybean (Cook *et al.*, 2012). Positive selection on the dosage effect of newborn duplicates is predicted in cases where the protein products physically interact with molecules such as toxins or nutrients, or in cases in which proteins need rapid and constant production at high levels (Kondrashov *et al.*, 2002; Kondrashov, 2012). The dosage effect of gene duplication might consequently be important for the establishment of a C_4_ cycle. Current models of C_4_ evolution hypothesize that a weak C_4_ cycle can first emerge using enzymes that have not been adapted to the C_4_ catalytic context (Sage, 2004; Heckmann *et al.*, 2013; Christin and Osborne, 2014; Mallmann *et al.*, 2014; Heckmann, 2016; Dunning *et al.*, 2017). Gene duplications increasing the transcript abundance of C_4_-related genes in plants with a weak C_4_ cycle would increase the strength of the pathway, which is predicted to boost carbon assimilation and fitness (Heckmann *et al.*, 2013; Mallmann *et al.*, 2014), leading to the preferential retention of the duplicates. We propose here to test the hypothesis that gene duplications contributed to the initial emergence of a C_4_ biochemistry via dosage effects, with subsequent neofunctionalization. We capitalize on the diversity of C_4_ enzymes that evolved in the recent past within the grass genus *Alloteropsis*.

The *Alloteropsis* genus contains five species, four of which are C_4_, while the fifth, *A. semialata*, encompasses C_4_ as well as non-C_4_ populations with and without a weak C_4_ cycle (Ellis, 1974; Lundgren *et al.*, 2016). The diversification of *A. semialata* took place during the last 3 million years (Lundgren *et al.*, 2015), and only a few genes are markedly up-regulated in the C_4_ accessions compared with C_3_ populations (Dunning *et al.*, 2017). In some cases, the identity of genes used for the C_4_ cycle differs among C_4_ populations of *A. semialata*, which is interpreted as the footprint of a gradual adaptation of C_4_ photosynthesis during the diversification of the group involving secondary gene flow among previously isolated populations (Olofsson *et al.*, 2016; Dunning *et al.*, 2017). This group therefore represents an outstanding system to investigate the small-scale processes that led to C_4_ photosynthesis, including the importance of genomic rearrangements such as duplications for C_4_ evolution.

Genome scans coupled with genome size estimates are used here to assess the gene content of accessions of the genus *Alloteropsis* varying in their photosynthetic type, testing (i) whether the copy number of genes encoding C_4_-related proteins varies among accessions of *Alloteropsis*; (ii) whether gene duplications coincide with the co-option of genes for a C_4_ function; and (iii) whether increases in gene copy number result from the duplication of genomic material or from retroposition events (i.e. insertion of retrotranscribed RNA into the genome; Kaessmann *et al.*, 2009). In addition, we retrieve published transcriptomes for members of the *Alloteropsis* genus (Dunning *et al.*, 2017) and associate them with newly generated high-coverage genome sequencing to test (iv) whether recently duplicated genes are expressed; (v) whether multiple copies all contribute to overall transcript abundance; and (vi) whether increases in copy number of C_4_-related genes along the phylogenetic tree were associated with increases in expression levels. This comparative analysis of gene copy numbers provides evidence for a potential role for recent gene duplications in physiological innovation through rapid and drastic changes of transcript abundance.

## Materials and methods

### Taxon sampling and genome data

A total of 20 genome-wide, low-coverage sequencing data sets of *Alloteropsis* J. Presl were retrieved from published studies (Table 1; Lundgren *et al.*, 2015; Olofsson *et al.*, 2016; NCBI accession no. SRP082653). These include two accessions of the C_4_*A. angusta* Stapf, one of the C_4_ species *A. cimicina* (L.) Stapf, and 17 of *A. semialata* (R. Br.) Hitchc. Among these 17 *A. semialata*, 12 are C_4_ individuals sampled across a broad geographical range from West Africa to Australia, and the five non-C_4_ include three individuals with a weak C_4_ cycle (‘C_3_+C_4_’ in Dunning *et al.*, 2017; note that this term is equivalent to ‘type II C_3_–C_4_ intermediates’ *sensu*Edwards and Ku, 1987) and two C_3_ individuals from South Africa. Each of the genomic data sets consists of paired-end Illumina reads, with read lengths of 100, 125, or 150 bp (Table 1). In this study, the raw reads were filtered using the NGSQC Toolkit (Patel and Jain, 2012) to retain only high-quality sequences (i.e. >70% of read length with Phred quality >20), and to remove primer and adaptor contaminated reads. The genome size and ploidy level of some of the individuals analysed here were retrieved from previous studies that used the same accessions (Lundgren *et al.*, 2015; Olofsson *et al.*, 2016). Some accessions were only available as herbarium samples, preventing estimates of genome sizes or ploidy levels.

**Table 1.** Genome data information

ID	**Species**	**Carbon** **isotope**	**Genome size** **(Gb/2Cx** ^***a***^ **)/ploidy**	**Country**	**Transcriptome sample** ^***b***^	**Sequencing** **batch** ^***c***^	**Sequencer**	**Read** **length**	**Total nuclear** **genome reads**	**Organellar** **reads (%**)^***d***^	**Theoretical** **coverage** ^***e***^
Cim1	*A. cimicina*	C_4_	–	Madagascar	ACIM	2	HiSeq 2500	100	20898025	2.0	0.95
Ang1	*A. angusta*	C_4_	–	DRC	–	5	HiSeq 3000	150	14751007	2.6	1.01
Ang2	*A. angusta*	C_4_	1.95/2*n*	Uganda	–	2	HiSeq 2500	100	18665954	1.9	0.96
RSA1	*A. semialata*	C_3_	–	South Africa	–	1	HiSeq 2500	100	14821009	0.8	0.67
RSA2	*A. semialata*	C_3_	1.80/2*n*	South Africa	RSA5	1	HiSeq 2500	100	12524356	0.6	0.70
TAN1	*A. semialata*	C_3_+C_4_	1.88/2*n*	Tanzania	TAN1	2	HiSeq 2500	100	18899157	4.0	1.01
TAN2-A	*A. semialata*	C_3_+C_4_	2.19/2*n*	Tanzania	TAN2	2	HiSeq 2500	100	20065838	4.2	0.92
TAN2-A^f^	*A. semialata*	C_3_+C_4_	2.19/2*n*	Tanzania	TAN2	6	HiSeq 2500	250	45774384	3.4	5.05
TAN3	*A. semialata*	C_3_+C_4_	–	Tanzania	–	3	HiSeq 2500	125	35782290	1.6	2.03
DRC1	*A. semialata*	C_4_	–	DRC	–	5	HiSeq 3000	150	33933832	3.6	2.31
DRC2	*A. semialata*	C_4_	–	DRC	–	4	HiSeq 3000	150	23098686	3.1	1.57
DRC3	*A. semialata*	C_4_	–	DRC	–	3	HiSeq 2500	125	28889427	6.4	1.64
DRC4	*A. semialata*	C_4_	–	DRC	–	5	HiSeq 3000	150	14749392	4.0	1.01
TAN4	*A. semialata*	C_4_	2.01/2*n*	Tanzania	TAN4	2	HiSeq 2500	100	18596076	3.2	0.93
RSA3	*A. semialata*	C_4_	5.22/6*n*	South Africa	RSA3	1	HiSeq 2500	100	13824190	0.8	0.26
KEN1	*A. semialata*	C_4_	–	Kenya	–	3	HiSeq 2500	125	25405608	4.9	1.44
BUR1	*A. semialata*	C_4_	1.95/2*n*	Burkina Faso	BUR1	1	HiSeq 2500	100	13498418	0.9	0.69
MAD1	*A. semialata*	C_4_	2.05/2*n*	Madagascar	MAD1	1	HiSeq 2500	100	16440692	1.8	0.80
THA1	*A. semialata*	C_4_	–	Thailand	–	2	HiSeq 2500	100	16873534	2.1	0.77
TPE1-3	*A. semialata*	C_4_	1.87/2*n*	Taiwan	TPE1	2	HiSeq 2500	100	15435339	4.8	0.83
TPE1-10^f^	*A. semialata*	C_4_	1.87/2*n*	Taiwan	TPE1	7	HiSeq 2500	250	169555422	3.4	21.92
AUS1	*A. semialata*	C_4_	2.20/2*n*	Australia	AUS1	1	HiSeq 2500	100	11600487	0.8	0.53

^*a*^ Genome size (Gb/2Cx)=total genome mass (pg)×0.978.

^*b*^ Data retrieved from Dunning *et al.* (2017).

^*c*^ Accessions with the same batch number were sequenced together.

^*d*^ Percentage of reads mapping to chloroplast and mitochondrial genomes.

^*e*^ Based on 2C genome size; after removing organellar reads; assuming a value of 2.2 Gb (maximum value of a diploid individual of *Alloteropsis*) for unknown genome sizes.

^*f*^ Data set generated for this study. Other data sets were retrieved from either Lundgren *et al.* (2015) or Olofsson *et al.* (2016).

High-coverage sequencing data sets were generated here for two individuals to allow single nucleotide polymorphism (SNP) analyses (see below). This included one C_3_+C_4_ accession from Tanzania (TAN2) already sequenced at low coverage and one C_4_ accession from a population where another individual was sequenced at low coverage (TPE1; Table 1). For these two samples, 250 bp long paired reads were obtained with the Illumina technology.

The different sequence data sets were obtained from whole genomic DNA, so that reads can belong to any of the nuclear, chloroplast, and mitochondrial genomes. Reads from the two organellar genomes were identified by mapping the genomic data sets onto representative chloroplast and mitochondrial genomes using Bowtie2 (Langmead and Salzberg, 2012) with default parameters, and removed before analyses. Mitochondrial genomes were assembled *de novo* (Supplementary text S1 at *JXB* online) using the approach described in Lundgren *et al.* (2015), while chloroplast genomes were retrieved from Lundgren *et al.* (2015) and Olofsson *et al.* (2016). On average, 3% of the initial reads were removed because of their organellar origin (Table 1).

### Mapping of reads on reference data sets

**Fig. 1.**
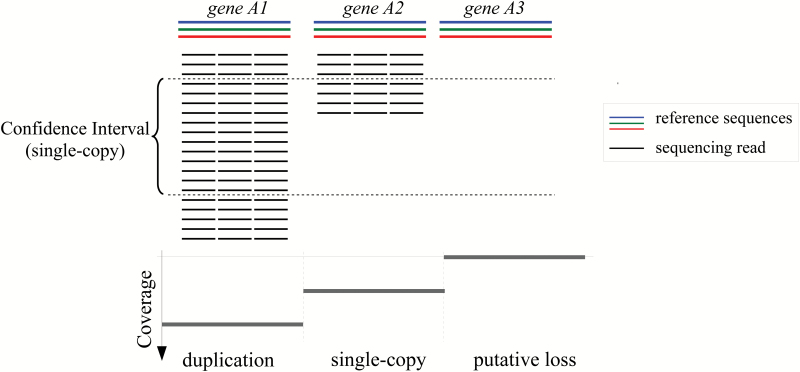
Read depth approach for gene copy number estimation. Duplications are inferred when the number of read counts expected for a determined gene is significantly higher than the expected read counts for single-copy genes, according to an underlying statistical model.

Gene copy numbers were estimated using a modified read depth approach (Alkan *et al.*, 2009; Yoon *et al.*, 2009; Teo *et al.*, 2012). This strategy divides the genome into non-overlapping regions (bins) and uses the number of genomic reads mapped to each of these regions to estimate gene copy number. Bins receiving in some accessions more or fewer reads than expected under a null statistical model are considered copy number variants (Fig. 1). Given the current lack of a reference genome for any *Alloteropsis* species, genomic data were mapped to a reference data set consisting of coding sequences (CDSs) of *A. cimicina* and *A. semialata*, which was retrieved from the transcriptome study of Dunning *et al.* (2017). Briefly, this data set comprises groups of co-orthologues at the Panicoideae subfamily level, the group of grasses that includes the genus *Alloteropsis*. Each group of co-orthologues encompasses all the genes that are descended by speciation and/or gene duplication from a single gene in the common ancestor of Panicoideae. Only genes captured in one of the *Alloteropsis* transcriptomes and with co-orthologues in at least one of *Sorghum bicolor* and *Setaria italica* were included. Increases in copy number detected here therefore correspond to duplications that happened after the initial diversification of Panicoideae, about 30 million years ago. Manually curated alignments using longer transcripts of 23 gene families with a known function in C_4_ biochemistry (Bräutigam *et al.*, 2011) and the gene encoding the Rubisco small subunit (*rbcS*) were added into the reference data set. These manually curated alignments improved read mapping accuracy in cases where paralogues with high sequence similarity were present, such as laterally acquired forms previously identified for phosphoenolpyruvate carboxylase (PEPC; *ppc* gene) and phosphoenolpyruvate carboxykinase (PCK; *pck* gene; Christin *et al.*, 2012; Dunning *et al.*, 2017). Overall, this genome-wide data set comprised 12688 groups of co-orthologues, belonging to 5589 gene families.

Genomic reads were mapped onto the genome-wide CDS data set using Bowtie2, with default parameters, randomly assigning reads mapped to multiple sequences to one of the top hits, and using the local alignment option. Reads were mapped as single-end reads to avoid false negatives when one of the reads mapped outside the CDS. The number of mapped reads (counts) per group of co-orthologues was obtained using SAMtools (Li *et al.*, 2009) and used to compute gene copy number estimates as described below.

### Estimates of copy numbers

Under the assumption that each site in the genome has an equal probability of being the first site of a given read, the expected read count (*c*) for any genomic region *i* of length *L* can be computed as:

$$\text{E}\left(c_{i}\right)=N\left(L_{i}/G\right)$$ (1)

where *N* is the total number of sequencing reads and *G* is the haploid genome size (in number of bases). Assuming the counts *c* is a random variable that follows a binomial distribution, with the total binomial trials being the total number of reads *N*, the probability of a region *i* being captured by one read is equivalent to the probability of success in each binomial trial, which is:

$$P=L_{i}/G$$ (2)

A well-known complication of quantitative genomic studies based on read depth is the sequencing bias linked to the GC content of the sequenced region, which is particular to sequencing approaches where library preparation includes PCR steps, as required for degraded DNA extracted from herbarium samples (Dohm *et al.*, 2008; Aird *et al.*, 2011; Benjamini and Speed, 2012; Teo *et al.*, 2012). The relationship between sequencing depth and GC content can vary across sequencing runs (Benjamini and Speed, 2012), and previous studies have quantified this relationship using various metrics (Alkan *et al.*, 2009; Bellos *et al.*, 2012; Benjamini and Speed, 2012). In this study, preliminary analyses confirmed that the relationship varied among the different batches of library preparation and sequencing (Supplementary Fig. S1). The relationship between read counts and GC content was consequently estimated for each sample by using the counts of genes extracted from the genome-wide reference mapping. Read counts were normalized by gene length, and genes with no count or counts >1.5 times the median count were removed from this particular analysis, to enrich the data set with putative single-copy genes. These length-normalized counts were then expressed as a linear function of the mean GC content of the target genes (*x*_*i*_), so that:

$$c_{i}/L_{i}=a+bx_{\text{i}}$$ (3)

The coefficients *a* and *b* were estimated individually for each genome data set using a linear model fit procedure in R (R Development Core Team, 2017). To homogenize the number of genes across GC content classes, 60 genes were randomly drawn from those present in each of nine equally spaced classes of GC content from 38% to 78%, and linear coefficients were calculated on the pooled subsample. Only genes longer than 700 bp were used here, since such long genes receive more reads and therefore provide more accurate copy number estimates. This procedure was repeated 100 times, providing a non-parametric estimate of variation for the coefficients. An approximate correction of the binomial probability of success in each trial (Equation 2) by the GC content was then obtained by substituting Equations 3 and 1 in Equation 2, so that:

$$P=L_{i}\times \left(a+bx_{\text{i}}\right)/N$$ (4)

Note that these new probabilities are independent of the genome size and can therefore be estimated for any sample. If E(*c*_*i*_) is the expected count when a target gene is present as a single copy, an estimate of the absolute number of copies *k*_*i*_ can be obtained as:

$$k_{i}=c_{i}/\text{E}\left(c_{i}\right)$$ (5)

The expected counts and confidence intervals for single-copy genes were computed using a binomial quantile function implemented in R, with a confidence level of 99% corrected for multiple comparisons using the Bonferroni method. Genes were considered duplicated if the counts were above the upper limit of this confidence interval, and single copy if the counts were within the confidence interval limits (inclusive). Although partial copies can exist following incomplete duplications, copy number estimates for duplicated genes were rounded up for follow-up analyses. Genes were considered absent when no read count was detected, provided the confidence intervals for the expected counts did not include zero. In such cases, and in cases where read counts were below the lower limit of the confidence interval, the genes were removed from the analysis, since accurate copy numbers could not be estimated.

### Quantitative real-time PCR estimates of copy number

A number of concerns have been raised about the use of high-throughput sequencing data for genome analyses of structural diversity, such as copy number variants (Benjamini and Speed, 2012; Teo *et al.*, 2012). In particular, the above-mentioned GC content bias and others resulting from the library preparations represent potential caveats. We consequently performed quantitative real-time PCR (qPCR) assays to confirm the accuracy of the copy numbers estimated from the genome data. The gene family encoding the key C_4_ enzyme phosphoenolpyruvate carboxylase (*ppc* genes) was selected for qPCR analyses since it included genes encompassing a wide range of copy numbers according to the read depth estimates (see the Results). Three paralogues (*ppc_1P3*, *ppc_1P6*, and *ppc_1P7*) were analysed in six individuals of *A. semialata* from a wide geographic and phylogenetic sampling (BUR1, RSA2, TAN2, TAN1, MAD1, and TPE1).

Alignments consisting of partial gene models of *ppc* groups of co-orthologues were assembled for *Alloteropsis* species using a genome-walking approach to include intron sequences, and were used as reference for primer design. Two pairs of primers per paralogue were designed to amplify 92–161 bp regions that include exon and intron sequences (except for one pair for *ppc_1P7*, which encompassed only exon sequences; Supplementary Table S1). The copy number estimated via qPCR consequently captured only putative duplications of genomic DNA, and excluded potential retroposition instances (Zhang, 2003; Kaessmann *et al.*, 2009; Reams and Roth, 2015). To perform the assays, genomic DNA (gDNA) was isolated from fresh leaves of *A. semialata* individuals using the DNeasy Plant Kit (Qiagen), following the manufacturer’s instructions. SYBR green-based qPCRs were prepared using 1× Power SYBR green PCR Master Mix (Thermo Fisher Scientific), 0.25 µM of each primer, and 6.25 ng of gDNA in a total volume of 20 µl, with three technical replicates and non-template controls per reaction. Assays were carried out on a QuantStudio 12K Flex Real Time PCR instrument (Life Technologies) with an initial incubation of 10 min at 95 °C (Taq activation), followed by 40 cycles of 15 s at 95 °C (denaturation) and 60 s at 60 °C (annealing and extension). Amplification specificity was assessed via melting curves generated immediately after each assay, in which samples were incubated for 15 s at 95 °C and 60 s at 60 °C, followed by incremental temperature increases of 0.3 °C up to 95 °C. The melting temperature of the amplified fragments was then calculated based on their expected sequences and compared with the peak temperature values obtained from the melting curve assays. Baseline, threshold cycle, and PCR efficiency were determined using the LinRegPCR software v. 2016.0 (Ramakers *et al.*, 2003). Samples with PCR efficiency <1.85 or >2.1 were excluded from the subsequent analysis. The Pfaffl method (Pfaffl, 2001) was used to correct for different PCR efficiencies across amplicon groups, and copy numbers of *ppc* genes were expressed relative to the mean of the two pairs of primers used for the *ppc_1P7* gene.

### Phylogenetic analyses of duplicated genes

To determine whether duplications of *ppc* and *pck* (see the Results) occurred before or after the diversification of *A. semialata* lineages, we assembled partial allele models by manually phasing polymorphisms using paired-end information. Ambiguous nucleotides were called for polymorphisms that could not be phased. Alleles of TPE1 and TAN2 were assembled using the high-coverage data, while raw transcriptome data of the genus *Alloteropsis* retrieved from Dunning *et al.* (2017) were used for the other accessions. Sequences were aligned using MAFFT v7.130b (Katoh and Standley, 2013), and phylogenetic trees were inferred using PhyML (Guindon and Gascuel, 2003) under a GTR+G model of nucleotide substitution, with 100 bootstrap pseudoreplicates.

### Allele-specific expression analyses

The relative contribution of each allele/paralogue of *pck* and *ppc* to the overall transcript abundance was assessed and compared with their relative frequency in the genomes through the analysis of SNPs. Reads from the genome and transcriptome data sets were mapped to reference alignments of the *ppc* and *pck* gene families, and the read depth was determined for each SNP of each gene using Geneious v. 6.8 (Kearse *et al.*, 2012). For each SNP, the abundance of the minor allele (defined on the transcriptome data as the variant base receiving fewer reads) was calculated as a proportion of the total read count for that site, for both transcriptome and genome data. Because the genomic frequency can vary among SNPs for multicopy genes (i.e. each variant can be present in any number of alleles up to twice the number of copies in a diploid individual), the contribution of different alleles to transcript abundance was evaluated via frequency correlations between transcriptome and genome data sets. Note that the polyploid individual was excluded from these analyses because of insufficient coverage to assess accurately polymorphisms among its high number of alleles.

### Association between changes in copy number and transcript abundance

To test for an association between changes in copy number and changes in gene expression, transcript abundances in leaves were retrieved for 14 C_4_-related genes captured in a study of transcriptomes of the genus *Alloteropsis* grown in controlled conditions (Dunning *et al.*, 2017). The average abundance between two biological replicates in reads per kilobase per million mapped reads (RPKM) is used here. Values were log10 transformed before analysis to homogenize variances. Accessions were considered for this analysis only if genome and transcriptome data were available for the same individual, or individuals from the same population, except in two cases (representing *A. cimicina* and the C_3_*A. semialata*) for which genome and transcriptome data were available for closely related individuals from different populations (Lundgren *et al.*, 2015; Olofsson *et al.*, 2016). Note that excluding these two individuals did not significantly alter the results. High-coverage sequence data were not used here to avoid pseudoreplication of some populations.

Homologous genes within a gene family do not represent independent data points as they result from events of gene duplication and/or speciation from a common ancestor. We consequently used phylogenetic generalized least squares (PGLS) under a Brownian model of evolution to test for correlated changes between gene copy number and transcript abundance using the R packages nlme and APE (Paradis *et al.*, 2004). A Bonferroni correction was used to adjust significance levels for multiple testing. The sequence alignment of the respective gene family was extracted from the genome-wide data set generated from transcriptomes (see above), and the accessions with no associated genome data were removed. Bayesian trees were inferred from this alignment under a GTR+G+I substitution model using MrBayes v3.2.2 (Ronquist *et al.*, 2012), with two parallel analyses running for 10000000 generations. After verifying the convergence of the runs, a consensus tree was generated using trees sampled after a burn-in period of 50%. The effect of topological uncertainty on the PGLS results was assessed by repeating the analysis using 100 independent trees sampled every 50000 generations after the burn-in period.

## Results

### Background distribution of gene copy numbers

Copy numbers were estimated for markers sampled across the genome for each accession, providing a background distribution of copy numbers per haploid chromosome set (Supplementary Fig. S2). Most genes were estimated as single copy, and the proportion of duplicated genes ranged from 9% to 28% across accessions, with 0.5–1.3% genes being absent (Table 2). The same copy numbers were estimated among individuals belonging to the same nuclear group, as previously defined in *A. semialata* (Olofsson *et al.*, 2016), for 82% of the genes, on average. Although there was a weak positive correlation between coverage and the proportion of absent genes (*R*^2^=0.34, *P*=0.055), no significant association was found between coverage and the proportion of single-copy (*R*^2^=0, *P*=0.41) or duplicated genes (*R*^2^=0, *P*=0.53), which suggests that the inferred duplications reflect biological rather than methodological differences. Similar estimates were found moreover between individuals from the same population based on low- and high-coverage data sets (Supplementary Fig. S3), indicating that low-coverage sequencing provides an accurate assessment of gene copy number variation. The variation in genome size (Table 1) was not explained by differences in gene copy number, with correlations being non-significant for the proportion of both absent and duplicated genes.

**Table 2.** Background distribution of gene copy numbers in *Alloteropsis* accessions

Accession	Species	Metabolism	Total genes analysed^*a*^	Proportions (%)^*b*^		
				Single-copy	**Duplicated**	**Absent**
Cim1	*A. cimicina*	C_4_	12057	89.4 (88.2–90.6)	9.8 (8.6–11)	0.9 (0.8–0.9)
Ang1	*A. angusta*	C_4_	8966	83.9 (81.4–86.9)	14.8 (11.7–17.4)	1.2 (1.2–1.4)
Ang2	*A. angusta*	C_4_	9700	84.2 (81.6–85.8)	14.5 (12.8–17.1)	1.3 (1.3–1.4)
RSA1	*A. semialata*	C_3_	8935	83.8 (81.7–85.9)	15.5 (13.4–17.6)	0.7 (0.7–0.8)
RSA2	*A. semialata*	C_3_	6996	86.4 (84.9–88)	13.1 (11.3–14.6)	0.5 (0.5–0.7)
TAN1	*A. semialata*	C_3_+C_4_	11376	88.4 (87.5–89.3)	10.8 (9.9–11.6)	0.8 (0.8–0.9)
TAN2	*A. semialata*	C_3_+C_4_	11221	86.1 (85.3–87.2)	13.2 (12.1–14.1)	0.7 (0.7–0.7)
TAN3	*A. semialata*	C_3_+C_4_	12195	79.5 (77.7–82.2)	19.9 (17.2–21.7)	0.6 (0.6–0.6)
DRC1	*A. semialata*	C_4_	12162	79 (76.4–81.3)	20.4 (18.1–23)	0.6 (0.6–0.6)
DRC2	*A. semialata*	C_4_	11946	81.1 (78.5–83.1)	18.3 (16.3–20.9)	0.6 (0.6–0.6)
DRC3	*A. semialata*	C_4_	11941	78.3 (75.3–80.7)	21 (18.6–24)	0.7 (0.7–0.7)
DRC4	*A. semialata*	C_4_	11014	81.4 (79.1–83.9)	17.9 (15.4–20.2)	0.7 (0.6–0.7)
TAN4	*A. semialata*	C_4_	11214	86.6 (85.6–87.3)	12.6 (11.8–13.6)	0.8 (0.8–0.8)
RSA3	*A. semialata*	C_4_	10248	88.1 (86.3–89.4)	11.2 (9.9–13.1)	0.6 (0.6–0.7)
KEN1	*A. semialata*	C_4_	10381	70.6 (64.1–76.5)	28.4 (22.5–35)	1 (1–1)
BUR1	*A. semialata*	C_4_	9448	88.4 (87.4–89.5)	10.9 (9.7–11.9)	0.7 (0.7–0.8)
MAD1	*A. semialata*	C_4_	10226	88.1 (86.7–89.1)	11.2 (10.2–12.6)	0.7 (0.7–0.7)
THA1	*A. semialata*	C_4_	10926	87.5 (86–88.6)	11.7 (10.6–13.3)	0.8 (0.7–0.8)
TPE1	*A. semialata*	C_4_	10730	88.5 (87.5–89.3)	10.7 (9.9–11.7)	0.8 (0.7–0.8)
AUS1	*A. semialata*	C_4_	7174	88.3 (87–89.7)	11 (9.6–12.3)	0.7 (0.6–0.7)

^*a*^ After removing genes having confidence intervals for the expected read counts that included zero, and/or read counts between 1 and the lower limit of the confidence interval (see the Materials and methods).

^*b*^ Percentage of single-copy, duplicated, or absent genes relative to the total number of genes analysed. Values are medians calculated from the resampling procedure used for the GC content correction, with the minimum and maximum values shown in parentheses.

### Duplications of C_4_ protein-coding genes

**Fig. 2.**
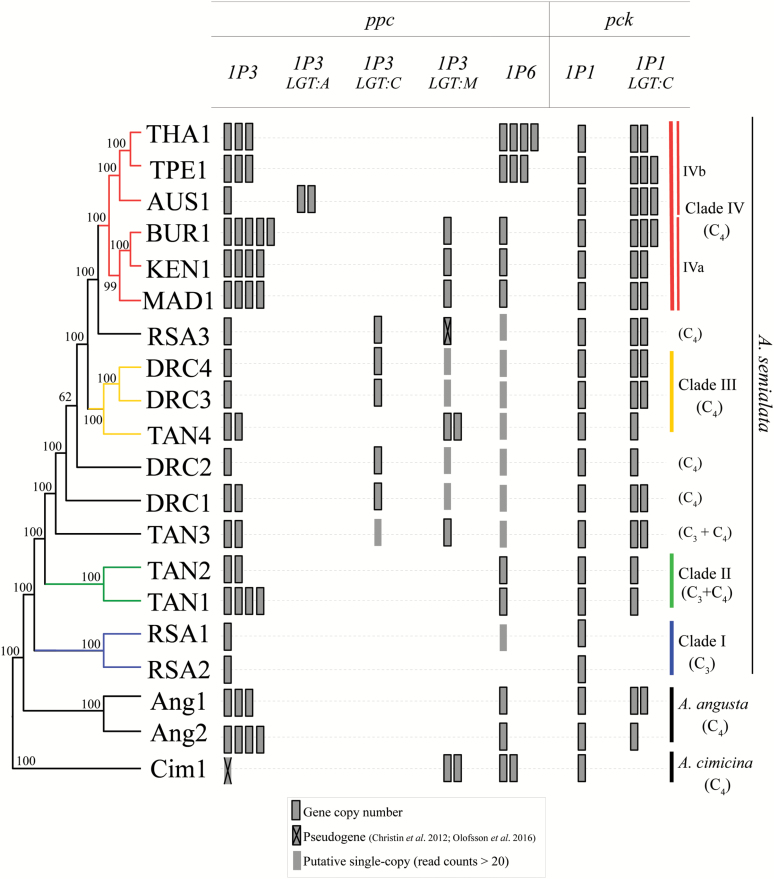
Copy number variation of selected genes of phosphoenolpyruvate carboxylase (*ppc*) and phosphoenolpyruvate carboxykinase (*pck*) in the *Alloteropsis* genus. *LGT:A*, *C*, and *M* are laterally acquired genes (Christin *et al.*, 2012). Nuclear phylogeny of the *Alloteropsis* genus was modified from Olofsson *et al.* (2016), with bootstrap support values shown near nodes, and lineages indicated on the right. Copy number estimates are based on low-coverage genome data, and are rounded to the nearest integer.

We estimated copy numbers for a total of 82 genes belonging to 23 gene families with some gene lineages encoding proteins known to be involved in the C_4_ pathway of some species. For 45 of these genes belonging to 19 families, at least one duplication was observed in the genus *Alloteropsis* (Supplementary Table S2). Putative ancient duplications (shared by *A. semialata*, *A. angusta*, and *A. cimicina*) include those for pyruvate kinase (*pk_1P1*) and NADP-dependent malic enzyme (*nadpme_1P4*). A number of genes have incurred independent duplications and/or secondary losses within *A. semialata* and *A. angusta*, including those for a tonoplast malate/fumarate transporter (*tdt_1P2*), in addition to those encoding phosphoenolpyruvate carboxylase (*ppc_1P3*) and phosphoenolpyruvate carboxykinase (*pck_1P1_LGT:C*). The *pck_1P1_LGT:C* gene was laterally acquired after the split between the C_3_ lineage and the lineage including C_3_+C_4_ and C_4_*A. semialata*, which now use it as part of their C_4_ cycle (Olofsson *et al.*, 2016; Dunning *et al.*, 2017), and subsequently duplicated only in the C_4_ group (Fig. 2). The *ppc* gene family has a particularly high diversity of copy numbers, which is especially marked for *ppc_1P3* and *ppc_1P6*, both of which are used for the C_4_ cycle of some accessions (Dunning *et al.*, 2017).

The phylogenetic distribution of duplicates could be explained by different combinations of duplications and secondary gene losses (Fig. 2), but these scenarios can be distinguished based on gene trees. The multiple copies of *pck_1P1_LGT:C* retrieved from the C_4_*A. semialata* form a monophyletic clade, which is split into subgroups corresponding to African and Asian/Australian accessions (Supplementary Fig. S4). This pattern could be explained by independent duplications in each of the two groups or a duplication at their base followed by recombination or concerted evolution within each of the groups. The multiple copies of *ppc_1P6* specific to TPE1 (and THA1; Fig. 2), which is the only accession to use this gene for its C_4_ pathway (Dunning *et al.*, 2017), are very similar and cluster in the phylogeny (Supplementary Fig. S5), which supports the hypothesis of very recent duplications. The multiple *ppc_1P3* copies of the C_3_+C_4_ and C_4_*A. semialata* form distinct, well-supported monophyletic groups and, within the C_4_ group, copies from the same accession tend to cluster despite a lack of resolution in some parts of the tree (Supplementary Fig. S6). This, again, suggests either independent duplications or concerted evolution following early duplications. Secondary losses of extra copies of *ppc_1P3* and the complete loss of *ppc_1P6* are inferred in the Australian accession (AUS1), which is the only accession carrying one of the laterally acquired *ppc* genes (*ppc_1P3_LGT:A;*Fig. 2).

**Fig. 3.**
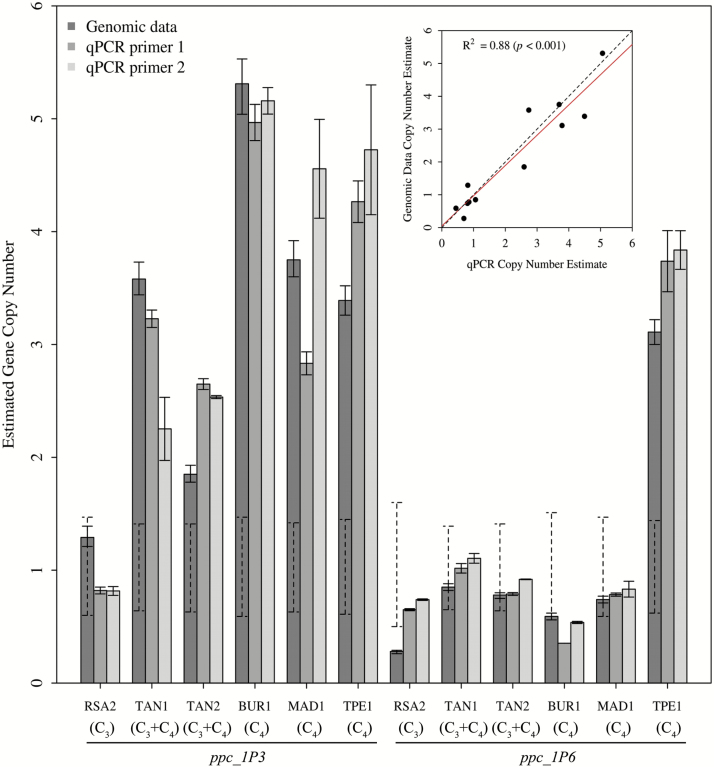
Comparison of copy number estimates obtained from qPCR assays and from low-coverage genomic data for the genes *ppc_1P3* and *ppc_1P6* in six *A. semialata* accessions. Copy numbers are expressed relative to the *ppc_1P7* gene. Error bars are SEs from 2–3 technical replicates for qPCR estimates, and non-parametric error estimates from the GC correction resampling procedure for the genomic estimates of copy number. Dashed lines on the genomic estimates indicate confidence intervals for single-copy genes. The upper panel indicates the correlation between qPCR estimates (mean value of both pairs of primers) and genomic estimates of copy number for *ppc_1P3* and *ppc_1P6*, with the red line being the regression line and the dashed line the identity line.

The copy numbers estimated for *ppc_1P3* and *ppc_1P6* from the genome data were significantly correlated with those estimated by qPCR (*R*^2^=0.88, *P*<0.001; Fig. 3). Since intronic regions were amplified in both pairs of primers used for the qPCR analysis, we conclude that the observed duplications correspond to duplications of genomic DNA. Differences in copy number of *ppc_1P3* between different primer pairs may be explained by the existence of a polymorphism in a region amplified by one of the primers, which would prevent the amplification of one of the alleles. Analyses of sequence alignments confirmed this was the case for at least one individual (MAD1). Alternatively, it is also possible that in other accessions some of the duplicates are present as partial copies originating from illegitimate recombination.

### Increases in transcript abundance associated with lineage-specific duplications

**Fig. 4.**
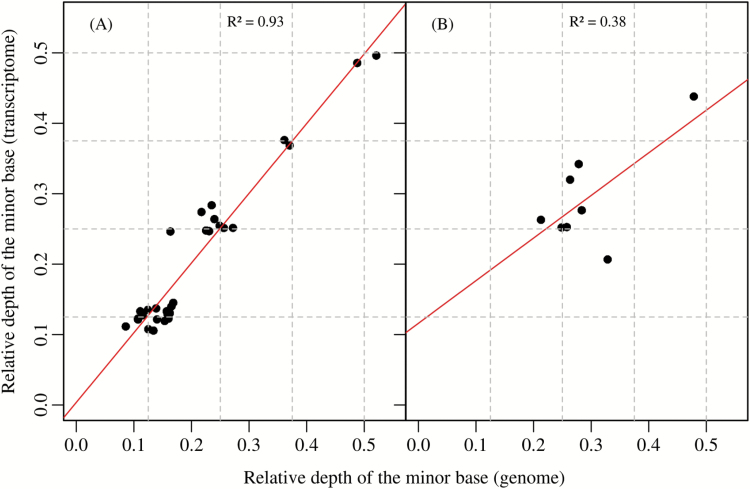
Relative read depth of variants detected at polymorphic sites of (A) *ppc_1P6* and (B) *ppc_1P3* genes in the genome and transcriptome of a C_4_ individual of *A. semialata* (TPE1). Each data point is a polymorphic site and is expressed as the depth of the minor base relative to the total depth for that site. The red line is the linear regression between transcriptome and genome data . The data points cluster around frequencies of 0.125, 0.25, 0.375, and 0.5, as indicated by dashed grid lines, which correspond to one, two, three, and four alleles out of a total of eight alleles from four duplicates.

Our analyses of C_4_-related genes revealed remarkable variation in copy number of *ppc* and *pck* among *Alloteropsis* lineages. For each polymorphic site, the frequency of the minor variant was strongly correlated between high-coverage genome and transcriptome data sets across the eight copies of *ppc_1P6* identified in TPE1 by the qPCR analysis (*R*^2^=0.93, *P*<0.001; Fig. 4). While the correlation between transcriptome and genome sequencing was also observed for *ppc_1P3* of TPE1, it was weaker (*R*^2^=0.38, *P*=0.06; Fig. 4; Supplementary Table S4), which might stem from lower overall transcript abundance and a small number of SNPs increasing statistical noise, or variation in the transcript contribution of different copies. The association between genome and transcriptome SNP frequencies varied among the other samples (Supplementary Table S4), which reflects a combination of low genome coverage of individual variants, variants not shared among the individuals used for genome and transcriptome sequencing, and biased transcriptome contribution of different copies. Nonetheless, the analyses of *ppc_1P3* and *pck_1P1_LGT:C* genes clearly show that multiple copies are expressed at consequent levels in the C_3_+C_4_ and C_4_ accessions, contributing to the elevated overall transcript levels of these genes in the C_3_+C_4_ and C_4_*A. semialata* (Supplementary Table S3; Dunning *et al.*, 2017). Overall, the SNP analyses provide strong support for duplicates being equally expressed in some accessions (e.g. *ppc_1P6* of TPE1), and show a widespread contribution of multiple copies to the elevated transcript abundance of *ppc* and *pck* genes.

**Fig. 5.**
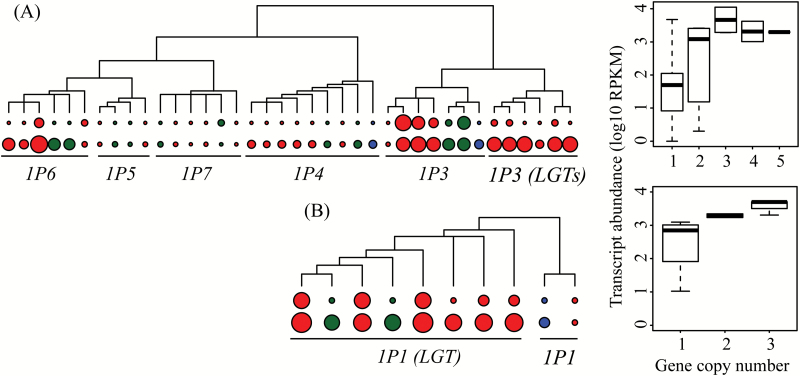
Association between changes in gene copy number and transcript abundance for (A) phosphoenolpyruvate carboxylase (*ppc*) and (B) phosphoenolpyruvate carboxykinase (*pck*). For each gene in each accession, circles next to the tips of the gene phylogeny are proportional to the estimated gene copy number (top) and transcript abundance (log10 RPKM; bottom). Circles are coloured according to the photosynthetic type (blue=C_3_, green=C_3_+C_4_, red=C_4_). The boxplots on the right show the distribution of transcript abundances per class of copy numbers for each gene family.

Finally, we tested whether the observed changes in copy number were statistically associated with changes in transcript abundance during the evolutionary diversification of the genus *Alloteropsis*. The conclusions of the statistical tests are robust to topological uncertainty (Supplementary Table S5), and we therefore discuss here only the results of the PGLS analyses based on the consensus tree (Table 3). Out of the 14 C_4_-related gene families for which transcript abundance was available in Dunning *et al.* (2017), 10 showed copy number variation among the accessions used for this analysis. We found a consistent positive association between changes in copy number and changes in transcript abundance that was significant after correction for multiple testing in two of them, *ppc* (*P*<0.001) and *pck* (*P*=0.002; Table 3; Fig. 5; Supplementary Fig. S7). In the case of *ppc*, these effects were mainly driven by a few copy number changes in *ppc_1P3* and *ppc_1P6* (Fig. 5A), which, along with the laterally acquired *ppc* genes (*ppc_1P3_LGT:A*, *ppc_1P3_LGT:M*, and *ppc_1P3_LGT:C*), are the most highly expressed copies of this gene family in the C_4_ accessions of the *Alloteropsis* genus (Dunning *et al.*, 2017). For *pck*, the duplication of *pck_1P1_LGT:C* after the split between the C_3_+C_4_ and C_4_ lineages was tightly associated with increases in transcript abundance of this gene (Fig. 5B). Although the other eight families include, in some cases, genes varying in copy number and transcript abundance, the statistical association was not significant after taking the phylogeny into account. In addition, analyses of *rbcS* showed a decrease in abundance in C_3_+C_4_ and C_4_ accessions, which was associated with increases in gene copy numbers, highlighting processes other than dosage effects during the diversification of this gene family in terms of copy number and transcript abundance (Supplementary Fig. S8).

## Discussion

### Recent gene duplications linked to physiological innovation via potential dosage effects

In this study, we used genome analyses to show that genes for *ppc* and *pck* recurrently increased in numbers during the evolution of C_4_ photosynthesis in the genus *Alloteropsis* (Fig. 2). These genes encode some of the few enzymes that reach very high levels in the C_3_+C_4_ and C_4_*A. semialata* (Ueno and Sentoku, 2006; Lundgren *et al.*, 2016; Dunning *et al.*, 2017), and increases in copy numbers statistically coincided with enhanced transcript abundance (Table 3; Fig. 5). One potential explanation for this pattern is that increased gene expression and high transcript abundance favoured frequent retroposition; that is, high transcription caused gene duplication (Kaessmann *et al.*, 2009). However, if this were the case, we would expect that increased copy number would uniquely involve exon sequences, which is disproved by our qPCR results. Analyses of polymorphisms further demonstrate that the multiple copies contribute to the overall high transcript abundances, with at least in some cases an equal contribution from each copy (Fig. 4). We therefore conclude that duplication of genomic DNA directly contributed to the expression levels of these genes, via dosage effects. Modifications of the regulatory mechanisms during the diversification of land plants and grasses are probably responsible for the variation of transcript abundance observed among single-copy gene lineages, and recent duplications would then have quickly enhanced the transcript level associated with some of the ancestral gene (Fig. 5), which can reach consequent levels in the non-C_4_ ancestors (Moreno-Villena *et al.*, 2018). Evidence for this mechanism was obtained here for only two genes, which encode proteins that are responsible for the initial fixation of atmospheric carbon into organic compounds and the release of CO_2_ to feed the C_4_ cycle, respectively. Three other enzymes show marked increases in transcript abundance in the C_3_+C_4_ and/or C_4_*A. semialata* (Dunning *et al.*, 2017), without evidence of gene copy number increases (Table 3). Unsurprisingly, the proposed dosage effect therefore concerns only a subset of the C_4_ genes, but it probably played a key role first in the emergence of a weak C_4_ cycle in the C_3_+C_4_ accessions, and then in the strengthening of this cycle in the C_4_ accessions, which is predicted to impact positively on fitness (Heckmann *et al.*, 2013; Mallmann *et al.*, 2014; Bräutigam and Gowik, 2016). Our results therefore suggest that dosage effects contributed to physiological innovation in the studied taxa, in association with changes in the regulatory properties of genes encoding other enzymes.

**Table 3.** Association between changes in gene copy number and changes in transcript abundance of C_4_-related gene families in *Alloteropsis*

Gene family	**Copy number** **range**	**Transcript abundance** **range** ^***a***^	***P*-value** ^***b***^
Alanine aminotransferase (ALA-AT)	1–2	0–1838	0.08
Aspartate aminotransferase (ASP-AT)	1–2	9–2632	0.48
Carbonic anhydrase (CA)	1–3	3–13169	0.46
Dicarboxylate transporter (DIT)	1	0–342	–
NAD-malate dehydrogenase (NAD-MDH)	1–4	21–1528	0.11
NAD-malic enzyme (NAD-ME)	1–2	12–162	0.57
NADP-malate dehydrogenase (NADP-MDH)	1	15–3537	–
NADP-malic enzyme (NADP-ME)	1–3	0–5746	0.56
PEP carboxykinase (PCK)	1–3	11–5187	**0.002**
PEP carboxylase (PEPC)	1–5	0–11153	**< 0.001**
Pyruvate phosphate dikinase (PPDK)	1–2	0–12796	0.82
PEP-phosphate translocator (PPT)	1–2	19–2593	0.62
Sodium bile acid symporter (SBAS)	1	17–7105	–
Triosephosphate-phosphate translocator (TPT)	1–2	8–3213	–

^*a*^ In RPKM; retrieved from Dunning *et al.* (2017);

^*b*^ *P*-values were obtained using a phylogenetic generalized least squares (PGLS) fitting under a Brownian model of character evolution; gene families lacking *P*-values do not show copy number variation, or contain representatives with no gene sequence available for the phylogenetic analysis.

Establishing the context of the duplications behind these increased copy numbers would require assembled genomes, but could involve unequal crossing over, chromosomal duplication, or the action of transposable elements (Zhang, 2003; Reams and Roth, 2015). Using high-coverage sequencing from genomic DNA or transcriptomes, we were able to assemble multiple copies of some *ppc* and *pck* genes in diploid accessions of *A. semialata*. While phylogenetic trees supported early duplications in some cases, the copies tended to group per accessions (Supplementary Figs S4–S6). The number of assembled copies was moreover below that estimated based on sequencing depth, suggesting that identical alleles exist. These patterns could be explained by recurrent gene duplications during the history of the *Alloteropsis* genus, or recombination, for example among tandem duplicates, leading to concerted evolution homogenizing the duplicated copies within geographically isolated lineages (Brown *et al.*, 1972; Nei and Rooney, 2005).

### Duplicates get lost after the acquisition of better-suited copies

At least three events of lateral gene transfers (LGTs) of *ppc* and one of *pck* occurred in the *Alloteropsis* genus (Christin *et al.*, 2012; Olofsson *et al.*, 2016), and some of the laterally acquired genes are expressed at high levels in the transcriptome of the accessions carrying such genes (Dunning *et al.*, 2017). In most of these accessions, the vertically inherited copies of *ppc* and *pck* are strongly down-regulated, or not expressed at all (Dunning *et al.*, 2017). Apart from the Southeast Asian clade, all C_4_ accessions of *A. semialata* studied here carry at least one laterally acquired *ppc* gene in their genomes. Interestingly, in this exception, multiple duplications of *ppc_1P6* were retained and are associated with drastic changes in transcript abundance that are specific to this clade (Fig. 5). On the other hand, the presence of some LGT copies (*ppc_1P3_LGT:C* and *ppc_1P3_LGT:A*) coincides with the loss of the initial duplicates of the vertically inherited *ppc_1P3* gene (Fig. 2; Olofsson *et al.*, 2016). These findings indicate that, once a gene better suited for the C_4_ function is acquired, the selective pressure on the original copy is relaxed, leading over time to pseudogenization and/or gene loss.

With multiple copies of genes related to C_4_ metabolism, the chances that some of these copies will acquire C_4_ adaptive mutations increase. Our analyses indeed identified non-synonymous polymorphisms among multiple copies of some genes. In four cases, such substitutions on *ppc* generate amino acid changes that were recurrently selected in a number of other C_4_ grasses, suggesting that they adapt the protein for the C_4_ catalytic context (Christin *et al.*, 2007). While not detectable with our approach, regulatory mutations, identified for other C_4_ groups (e.g. Gowik *et al.*, 2004; Akyildiz *et al.*, 2007), might similarly be present in only some of the multiple copies reported here. Genes that do not have the adaptive mutations can be lost via negative selection or drift, and those with the beneficial mutations are retained, leading to typical neofunctionalization. As reported here, the acquisition of more suitable gene versions, illustrated by the LGTs, can indeed relax the selection over duplicated copies that were once preserved via dosage selection, but from there on will be subjected to pseudogenization or eventually neofunctionalization. This suggests that during the course of evolution, fewer, more optimized genes are likely to remain, which would explain why more established C_4_ lineages are not enriched in C_4_-related genes (Williams *et al.*, 2012; van den Bergh *et al.*, 2014). The presence of multiple gene copies therefore probably contributes to the emergence of C_4_ photosynthesis via a combination of dosage effects and increased opportunities for neofunctionalization, both of which are evolutionarily transient.

### Low-coverage sequencing correctly identified duplicates

Low-coverage genomic data sets are increasingly used for a wide range of population genomic (Buerkle and Gompert, 2013; Nicod *et al.*, 2016; Olofsson *et al.*, 2016) and phylogenetic studies (Bock *et al.*, 2014; Dodsworth, 2015; Washburn *et al.*, 2015). While such data sets are relatively cheap to obtain and can be generated from poorly conserved samples such as those from museum collections (Besnard *et al.*, 2014; Silva *et al.*, 2017), they come with their limitations. In particular, sequencing biases are inherent to the PCR steps involved in the sample preparation, and lead to over-representation of regions with specific GC contents (Benjamini and Speed, 2012; Ross *et al.*, 2013; see the Materials and methods). It is therefore necessary to validate the results with independent evidence, provided here by qPCR. Slight variation between qPCR estimates and those based on low-coverage data confirmed that copy numbers inferred from read depths are in some cases under- or overestimated, as expected given both the low coverage and the difficulty in precisely correcting for the sequencing bias. However, the general patterns are correctly identified, as indicated by the similarity of estimates among closely related accessions, and by the strong agreement in the estimates based on low- and high-coverage data sets in cases where both were available for individuals from the same population (Supplementary Fig. S3). In addition, individual events of gene duplication inferred from low-coverage data are qualitatively correct, being in all cases confirmed by independent qPCR.

The intersection of different lines of evidence shows that our approach represents a valid strategy to infer patterns of copy number variation for a large number of non-model species. Some of the genomic data sets included here come from samples only available in herbarium collections, which were collected up to 60 years ago (Olofsson *et al.*, 2016). In cases where living material is not available, low-coverage sequencing represents a valuable resource to shed light on not only the phylogenetic relationships, but also the genomic content of important taxa (Besnard *et al.*, 2014), and, as shown here, variation in gene copy number. In the near future, the increasing availability of sequencing data sets for non-model species will offer multiple opportunities to track the genomic dynamics underlying a large array of physiological adaptations in a variety of taxa.

### Conclusion

Using comparative genomics, we showed that the duplication of genes encoding two key enzymes required for C_4_ photosynthesis coincided with the co-option of these genes for the new metabolic pathway. Based on published transcriptome data, we propose that changes in copy number altered the expression levels via pure dosage effects, with duplication events representing major effect mutations that can rapidly double transcription levels of some genes, which might have contributed to the emergence of a weak C_4_ cycle in some plants. Once the C_4_ cycle was in place, selection could act to optimize it, which probably involved fixing beneficial mutations on individual genes, including substitutions and indels in both regulatory and coding sequences. The selection of better-suited isoforms apparently led to pseudogenization of the previous duplicates. We therefore suggest that gene copy number decreases as beneficial mutations in the promoter or coding sequences are fixed, in a process of neofunctionalization. The beneficial effects of gene duplication for physiological innovation are therefore likely to be transitory, with no footprint on longer evolutionary scales.

## Supplementary data

Supplementary data are available at *JXB* online.

Fig. S1. Relationship between length-normalized read count and GC content in the genomic data sets of accessions of the genus *Alloteropsis*.

Fig. S2. Background gene copy number distribution in accessions of the genus *Alloteropsis*. Copy numbers are expressed as observed read count divided by expected read count.

Fig. S3. Comparison between copy number estimates using high- and low-coverage data sets for individuals within the same population.

Fig. S4. Phylogenetic tree of *pck* genes in the genus *Alloteropsis*.

Fig. S5. Phylogenetic tree of *ppc_1P6* genes in the genus *Alloteropsis*.

Fig. S6. Phylogenetic tree of *ppc_1P3* genes in the genus *Alloteropsis*.

Fig. S7. Distribution of transcript abundance among classes of gene copy numbers for 12 C_4_-related gene families.

Fig. S8. Distribution of transcript abundance among classes of copy numbers for genes encoding the small unit of Rubisco (*rbcS*).

Table S1. List of primer sequences of *ppc* genes used for quantitative real-time PCR assays.

Table S2. List of duplicated genes of C_4_-related gene families within the genus *Alloteropsis*.

Table S3. Read depth of transcriptome and genome data for polymorphic sites of *ppc* and *pck* genes of accessions of the genus *Alloteropsis*.

Table S4. Association between read depth of transcriptome and genome data for polymorphic sites of *ppc* and *pck* genes of *Alloteropsis* accessions.

Table S5. Effect of phylogenetic tree on the phylogenetic generalized least squares (PGLS) analysis used to test for an association between changes in gene copy number and changes in transcript abundance.

Text S1. Mitochondrial genome contigs of *Alloteropsis semialata* (accession MAD1).

Supplementary Figures Tables

Supplementary Table S3

Supplementary File S1
