# Supplementary material for: Gene duplication and dosage effects during the early emergence of C4 photosynthesis in the grass genus Alloteropsis
Source: J Exp Bot. 2018 Feb 1;69(8):1967–80. doi: 10.1093/jxb/ery029 (PMC6018922; doi:10.1093/jxb/ery029)
Supplement: Supplementary Figures Tables [file ery029_suppl_supplementary_figures_s1-s8_tables_s1_s5.pdf]

Supplementary Data for:

"Gene duplication and dosage effects during the early emergence of C_4 photosynthesis in the grass genus *Alloteropsis*"

Authors: Matheus E. Bianconi, Luke T. Dunning, Jose J. Moreno-Villena, Colin P. Osborne, Pascal-Antoine Christin

This supplementary information contains eight figures, five tables and a text file:

Fig. S1. Relationship between length-normalized read count and GC-content in the genomic datasets of accessions of the genus *Alloteropsis*. Black points fall in the interval presumably enriched in single-copy genes that were randomly resampled for the nonparametric error estimation of gene copy numbers, and gray points are the whole set of genes analysed in this study. Red lines represent the linear regression of length-normalized read count and GC-content for each set of resampled genes.

Fig. S2. Background gene copy number distribution in accessions of the genus *Alloteropsis*. Copy numbers are expressed as observed read count divided by expected read count.

Fig. S3. Comparison between copy number estimates using high coverage and low coverage datasets for individuals within the same population. The black dashed lines represent the linear regressions of low coverage (y) and high coverage estimates (x), and the red lines indicate identity. Coloured points are copy number estimates for the gene families *ppc* (green) and *pck* (blue).

Fig. S4. Phylogenetic tree of *pck* genes in the genus *Alloteropsis*. Colours indicate C_3 (blue), C_3+C_4 (green) and C_4 (red) accessions of *A. semialata*. Bootstrap support values are shown near branches when greater than 50.

Fig. S5. Phylogenetic tree of *ppc_1P6* genes in the genus *Alloteropsis*. Colours indicate C_3 (blue), C_3+C_4 (green) and C_4 (red) accessions of *A. semialata*. Bootstrap support values are shown near branches when greater than 50.

Fig. S6. Phylogenetic tree of *ppc_1P3* genes in the genus *Alloteropsis*. Colours indicate C_3 (blue), C_3+C_4 (green) and C_4 (red) accessions of *A. semialata*. Bootstrap support values are shown near branches when greater than 50.

Fig. S7. Distribution of transcript abundance among classes of gene copy numbers for 12 C_4-related gene families.

Fig. S8. Distribution of transcript abundance among classes of copy numbers for genes encoding the small unit of ribulose-1,5-bisphosphate carboxylase/oxygenase (Rubisco; *rbcS*).

Table S1. List of primer sequences of *ppc* genes used for quantitative real-time PCR assays.

Table S2. List of duplicated genes of C_4-related gene families within the genus *Alloteropsis*.

Table S3. Read depth of transcriptome and genome data for polymorphic sites of *ppc* and *pck* genes of accessions of the genus *Alloteropsis* (.xls file).

Table S4. Association between read depth of transcriptome and genome data for polymorphic sites of *ppc* and *pck* genes of *Alloteropsis* accessions.

Table S5. Effect of phylogenetic tree on the phylogenetic generalized least squares (PGLS) analysis used to test for an association between changes in gene copy number and changes in transcript abundance.

Text file: Mitochondrial genome contigs of *Alloteropsis semialata* (accession MAD1).

Fig. S1.

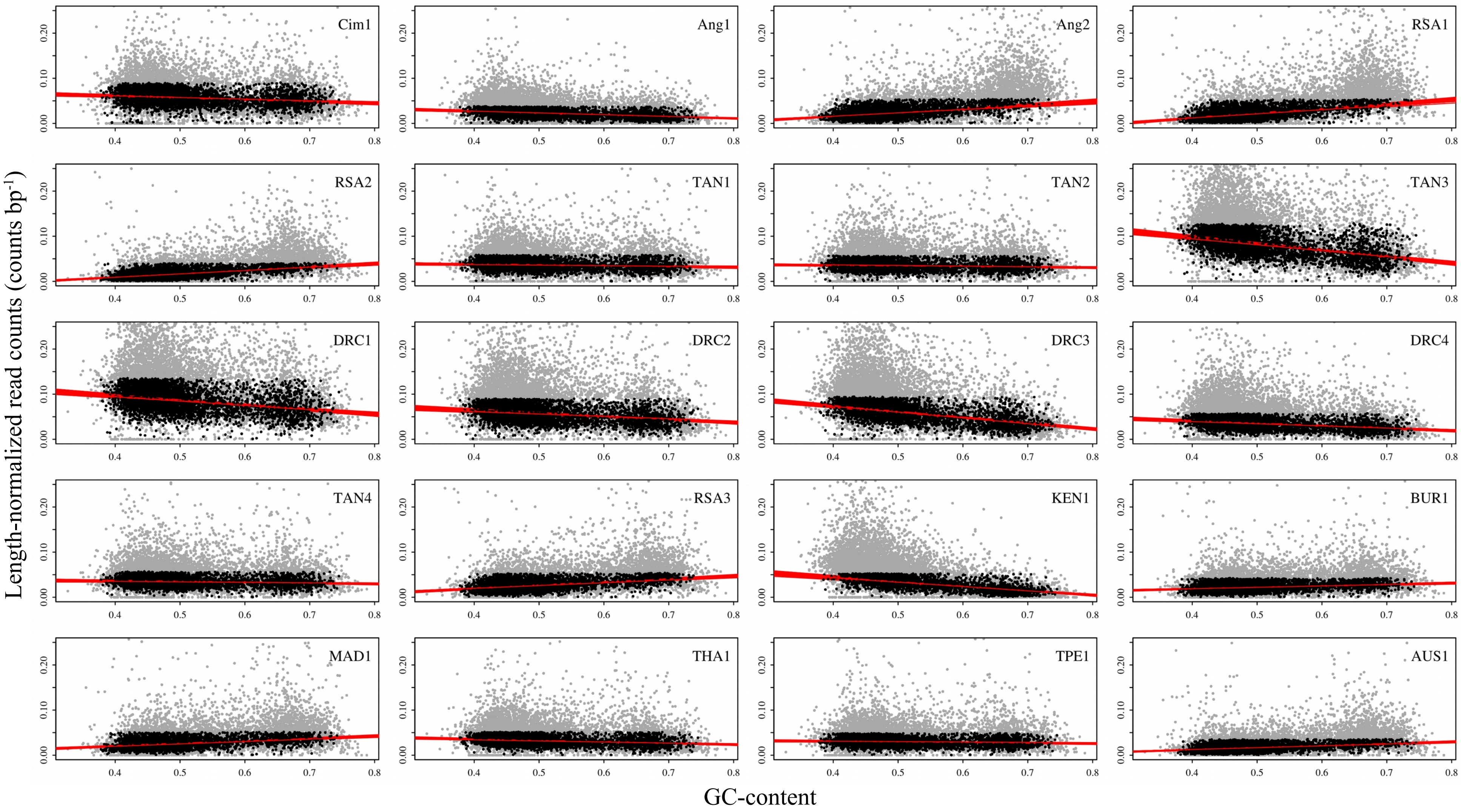
Cim1
Ang1
Ang2
RSA1
RSA2
TAN1
TAN2
TAN3
DRC1
DRC2
DRC3
DRC4
TAN4
RSA3
KEN1
BUR1
MAD1
THA1
TPE1
AUS1
Length-normalized read counts (counts bp-1)
GC-content
0.00
0.10
0.20
0.4
0.5
0.6
0.7
0.8

Fig. S2.

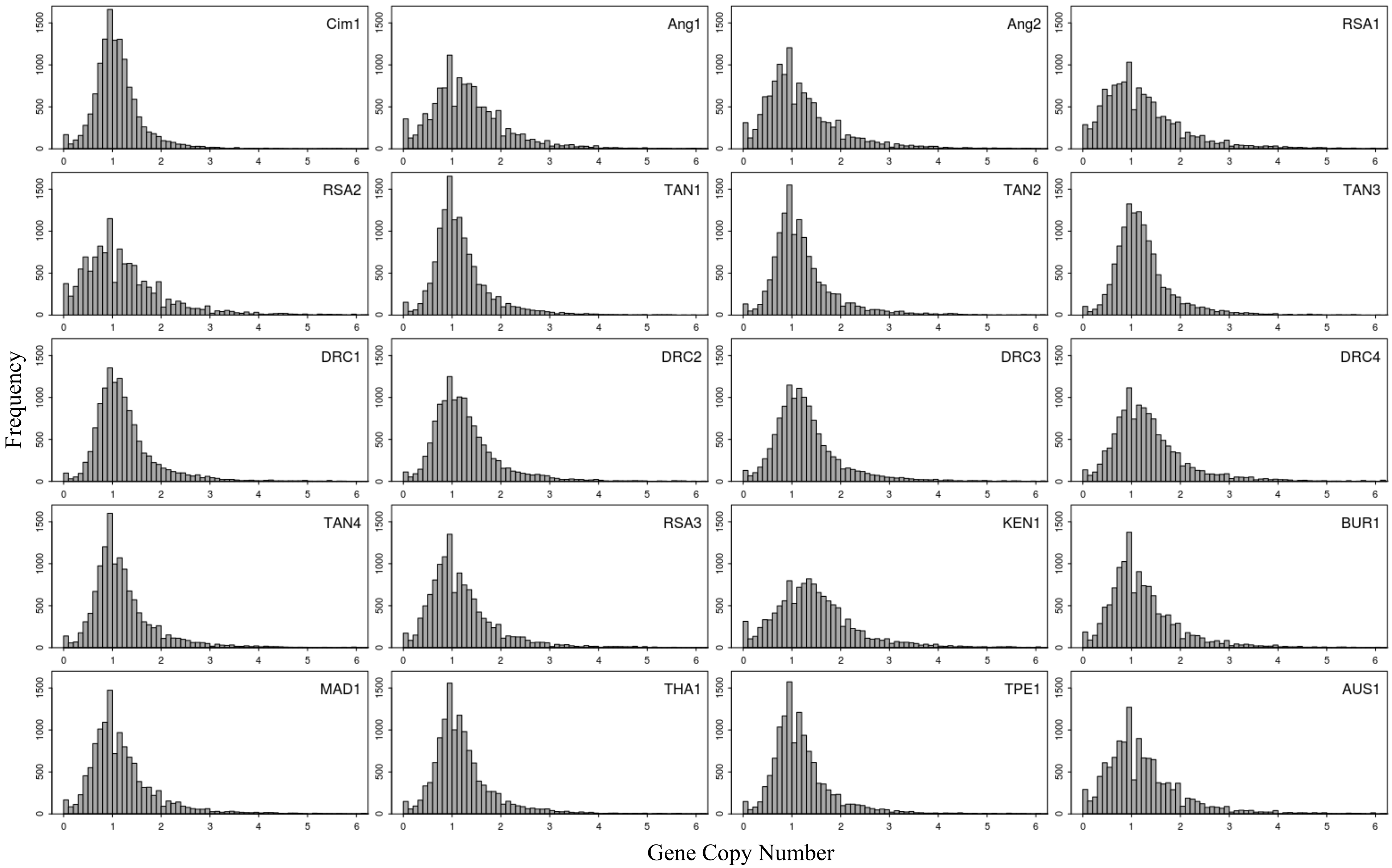
Cim1
Ang1
Ang2
RSA1
RSA2
TAN1
TAN2
TAN3
DRC1
DRC2
DRC3
DRC4
TAN4
RSA3
KEN1
BUR1
MAD1
THA1
TPE1
AUS1
Frequency
Gene Copy Number

Fig. S3.

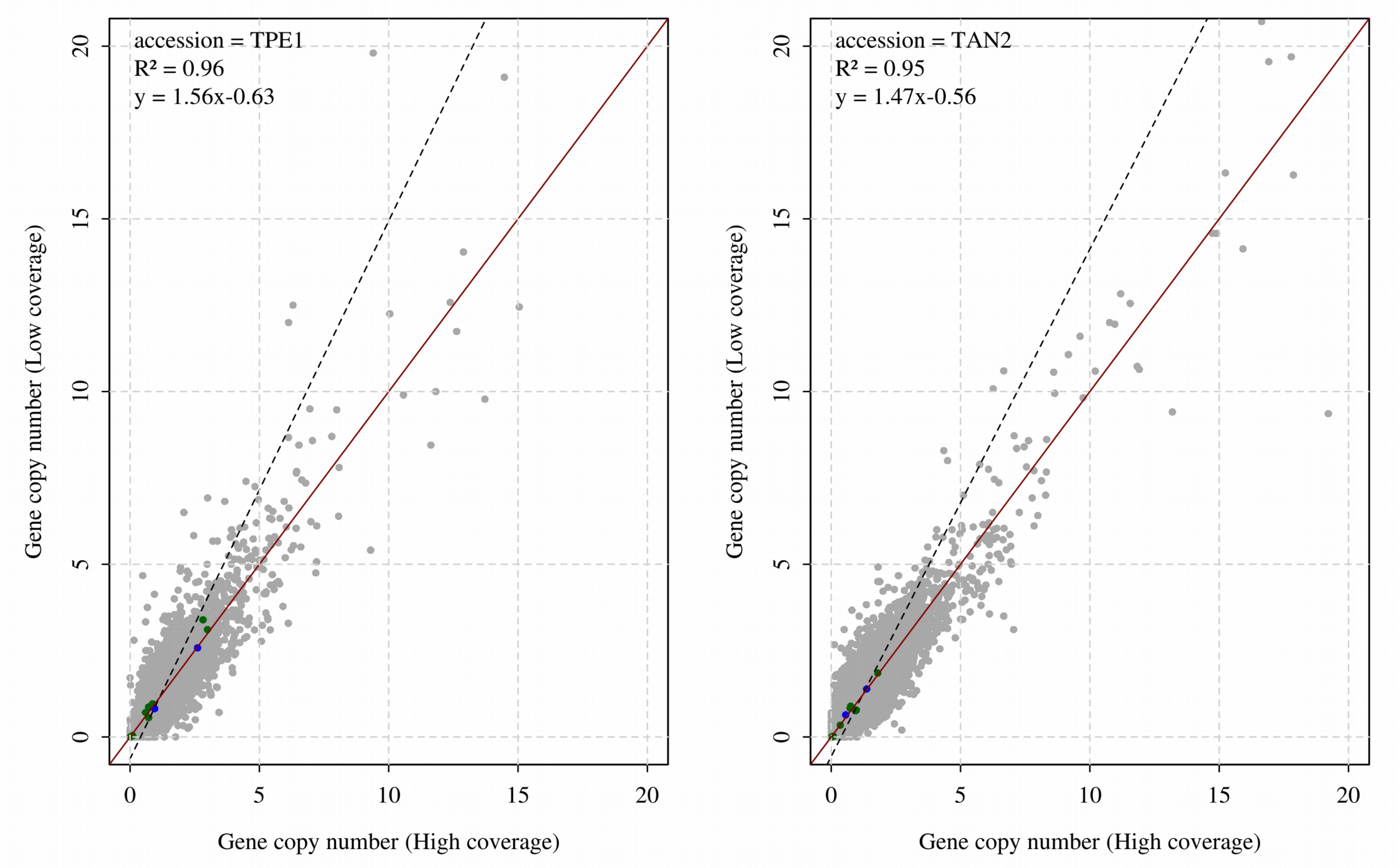

accession = TPE1
R² = 0.96
y = 1.56x-0.63
Gene copy number (Low coverage)
Gene copy number (High coverage)
0
5
10
15
20
accession = TAN2
R² = 0.95
y = 1.47x-0.56
Gene copy number (Low coverage)
Gene copy number (High coverage)

Fig. S4.

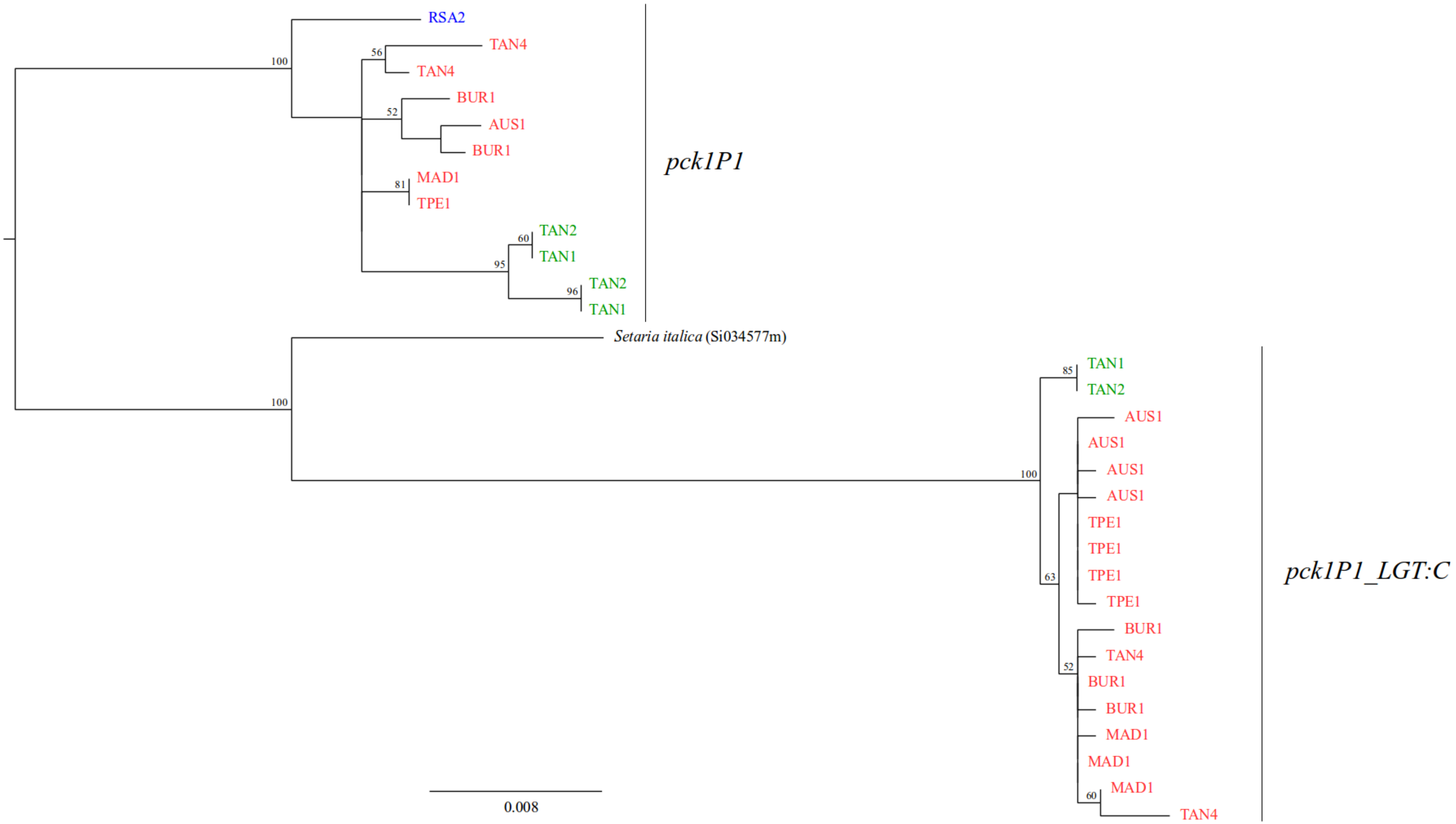

RSA2
TAN4
TAN4
BUR1
AUS1
BUR1
MAD1
TPE1
TAN2
TAN1
TAN2
TAN1
pck1P1
Setaria italica (Si034577m)
TAN1
TAN2
AUS1
AUS1
AUS1
AUS1
TPE1
TPE1
TPE1
TPE1
BUR1
TAN4
BUR1
BUR1
MAD1
MAD1
MAD1
TAN4
pck1P1_LGT:C
0.008
100
56
52
81
60
95
96
100
85
100
63
52
60

Fig. S5.

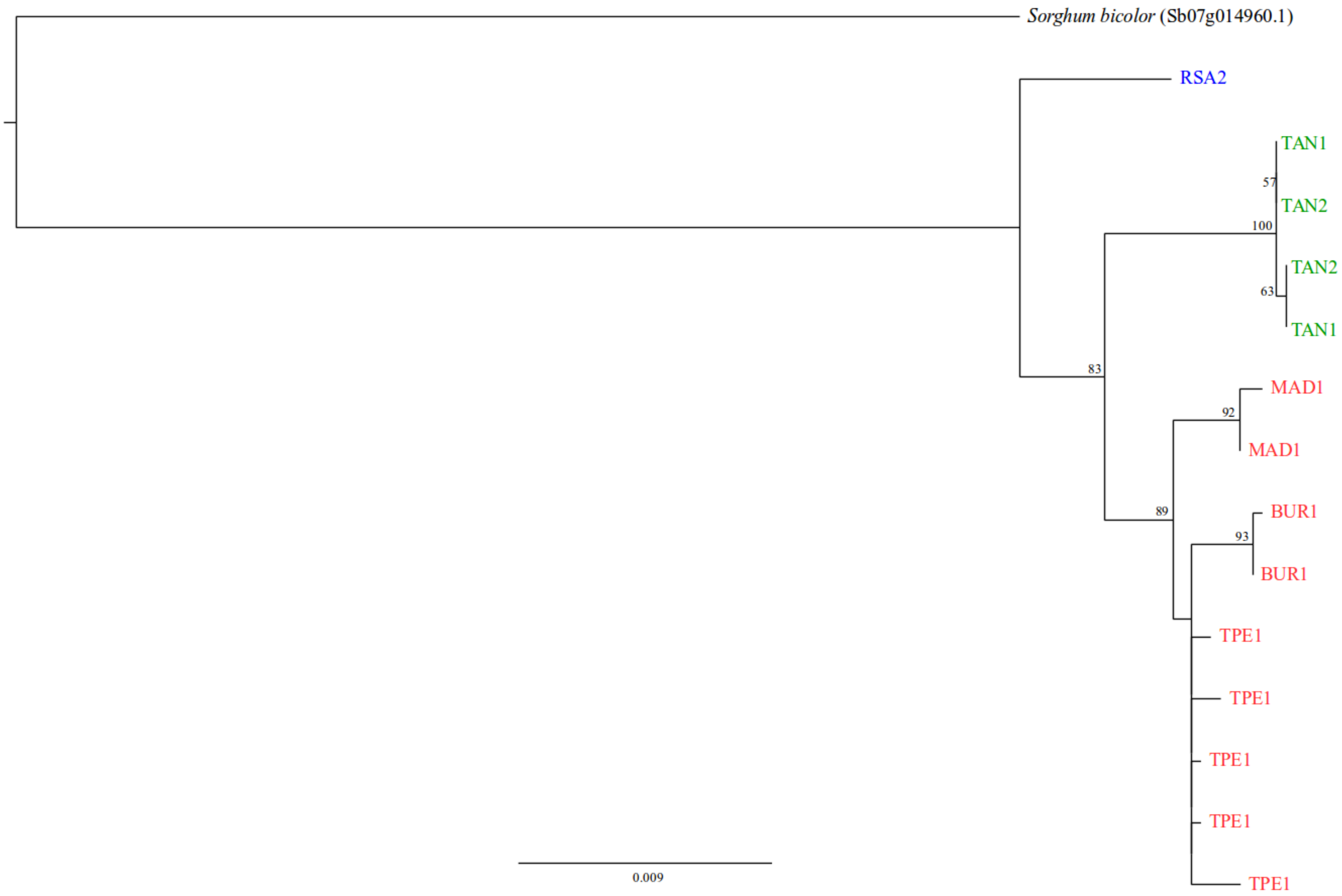
Sorghum bicolor (Sb07g014960.1)
RSA2
TAN1
57
TAN2
100
TAN2
63
TAN1
83
MAD1
92
MAD1
89
BUR1
93
BUR1
TPE1
TPE1
TPE1
TPE1
TPE1
0.009

Fig. S6.

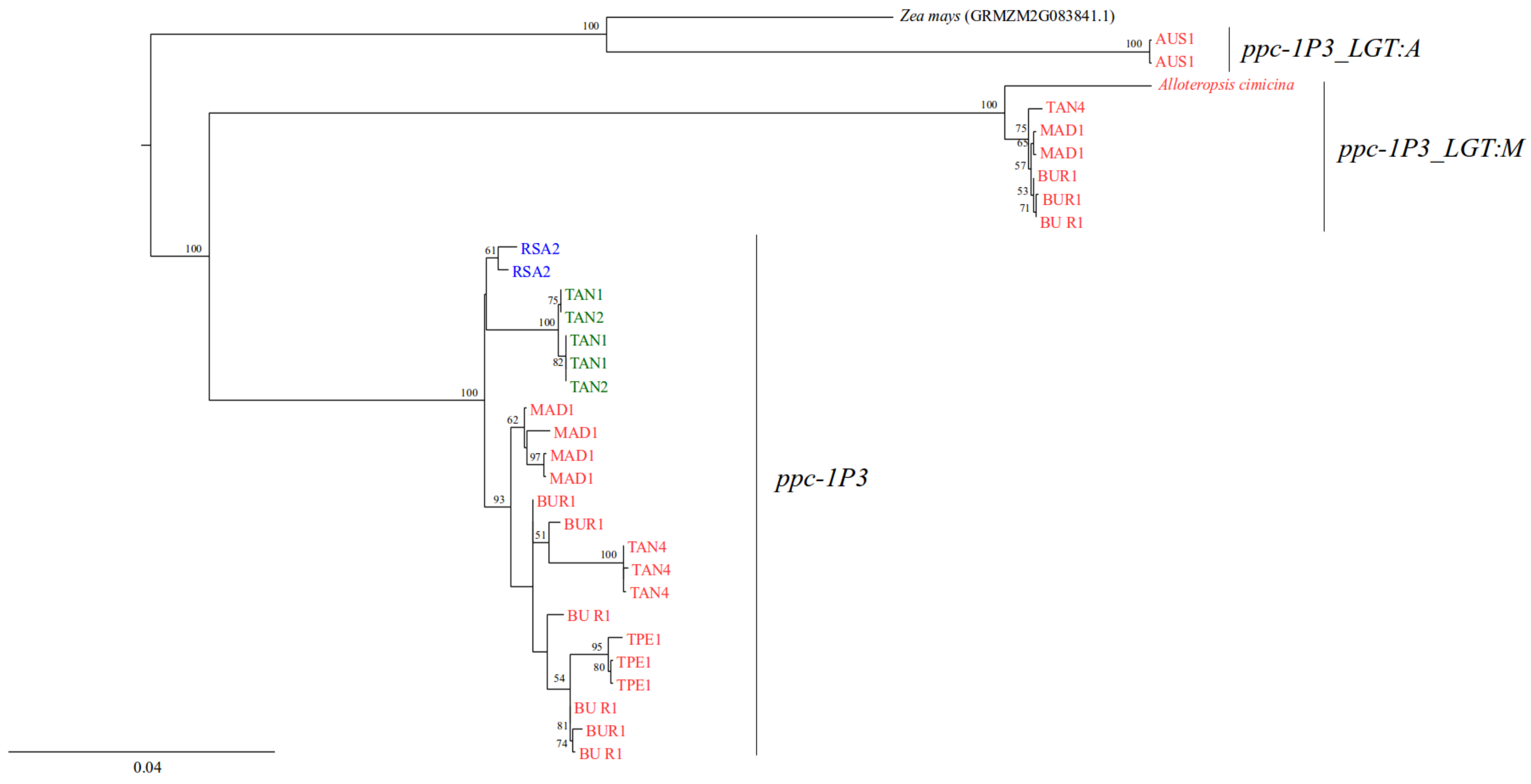

Zea mays (GRMZM2G083841.1)
100
100
AUS1
AUS1
ppc-1P3_LGT:A
Alloteropsis cimicina
100
TAN4
75
MAD1
65
MAD1
57
BUR1
53
BUR1
71
BU R1
ppc-1P3_LGT:M
100
61
RSA2
RSA2
75
TAN1
100
TAN2
TAN1
82
TAN1
TAN2
100
62
MAD1
MAD1
97
MAD1
MAD1
93
BUR1
51
BUR1
100
TAN4
TAN4
TAN4
BU R1
95
TPE1
80
TPE1
TPE1
54
BU R1
81
BUR1
74
BU R1
ppc-1P3
0.04

Fig. S7.

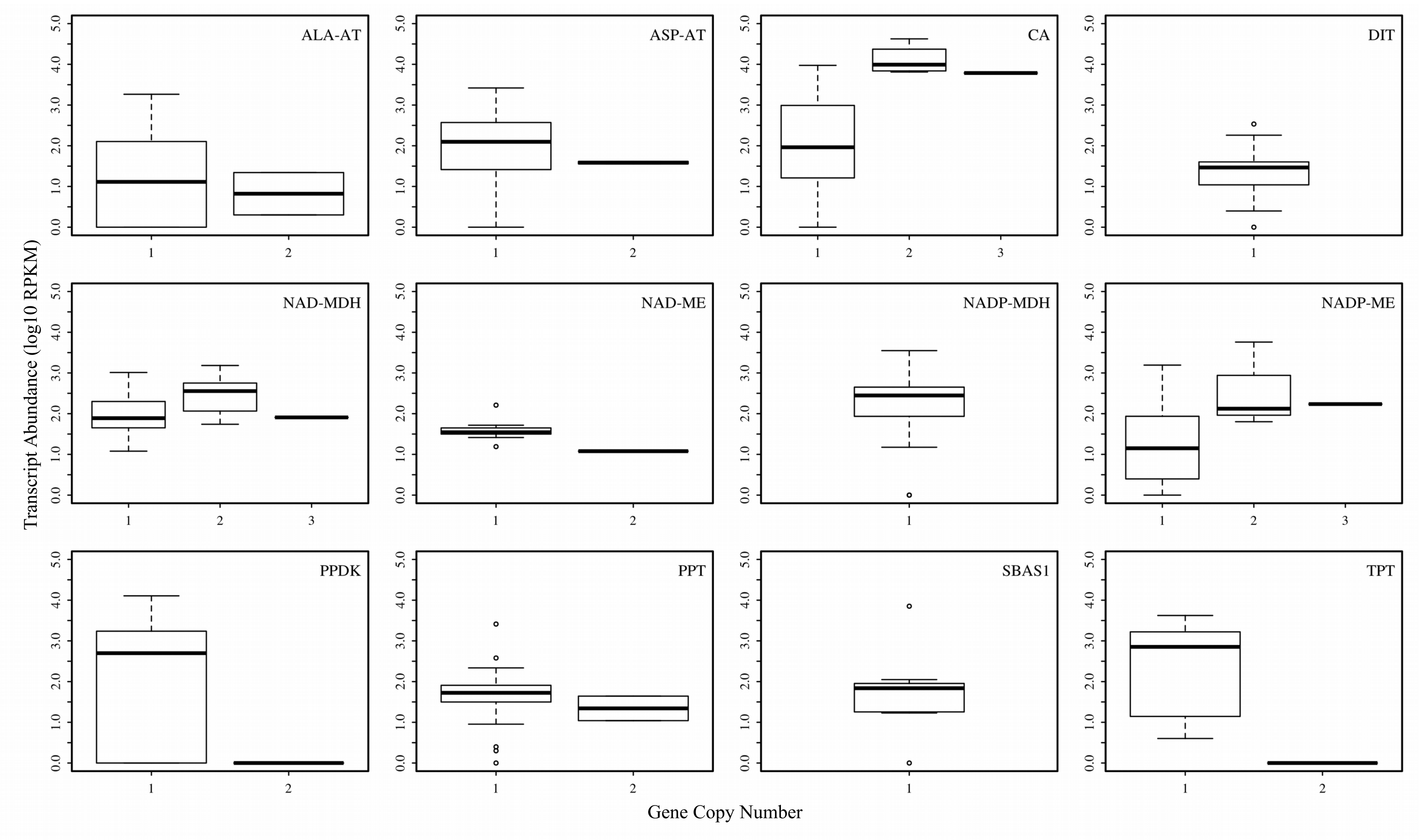

ALA-AT
ASP-AT
CA
DIT
NAD-MDH
NAD-ME
NADP-MDH
NADP-ME
PPDK
PPT
SBAS1
TPT
Transcript Abundance (log10 RPKM)
Gene Copy Number
0.0
1.0
2.0
3.0
4.0
5.0
1
2
3

Fig. S8.

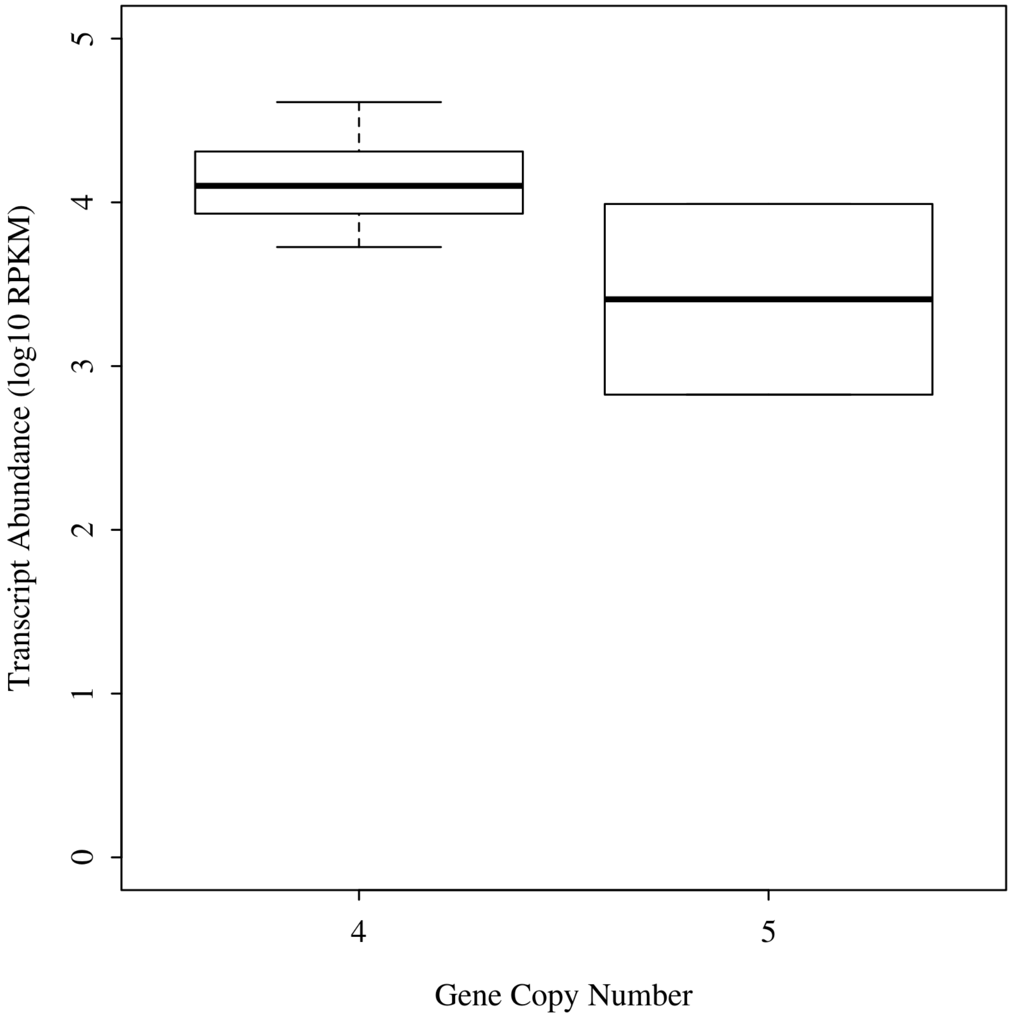

Transcript Abundance (log10 RPKM)
0
1
2
3
4
5
4
5
Gene Copy Number

Table S1. List of primer sequences of *ppc* genes used for quantitative real-time PCR assays.

Gene	Primer ID	Sequence	Expected amplicon length/ Melting temperature
ppc_1P6 - pair 1 (exon 6 – intron 6)	ppc1P6-FOR-p1<br>ppc1P6-REV-p1	5'-GCACGAGCAGATGAATTAC-3'<br>5'-GTGAAAGTAGCCATCTACCATG-3'	92 bp<br>73.8°C
ppc_1P6 - pair 2 (exon 7 – intron 7)	ppc1P6-FOR-p2<br>ppc1P6-REV-p2	5'-GCACGCCAGTTGTTATCCAG-3'<br>5'-CAATATGTGCAGTTCAAAGGTTC-3'	109 bp<br>76.4°C
ppc_1P3 - pair 1 (exon 8 – intron 8)	ppc1P3_native-FOR-p1<br>ppc1P3_native-REV-p1	5'-GTTCGTCGAGTACTTCCGATC-3'<br>5'-GTGTGGCCTGACACGATC-3'	115 bp<br>78.8°C
ppc_1P3 - pair 2 (exon 8 – exon 9)	ppc1P3_native-FOR-p2<br>ppc1P3_native-REV-p2	5'-CGGTTCGTCGAGTACTTCCG-3'<br>5'-CGTACTCCGTCTCAGGTGTGG-3'	136 bp<br>80.4°C
ppc_1P7 - pair 1 (exon 7 – intron 7)	ppc1P7-FOR-p1<br>ppc1P7-REV-p1	5'-CGTGTGATTCTGAGTGATGTC-3'<br>5'-GCTAGACAAATCGAATGACCAC-3'	161 bp<br>78.3°C
ppc_1P7 - pair 2 (exon 8)	ppc1P7-FOR-p2<br>ppc1P7-FOR-p2	5'-CCGACATACTGATGTTATGGA-3'<br>5'-ACGGCCTCTTTCCATTAAGTT-3'	119 bp<br>76.7°C

Table S2. List of duplicated genes of C_4-related gene families within *Alloteropsis*.

Gene family	Gene	*Alloteropsis* lineages with gene duplications[1]
Adenylate kinase (AK)	*ak_1P1*	I
	ak_2P2	I, IVa
Alanine aminotransferase (ALA-AT)	*alaat_1P2*	II, III, IVa
	alaat_1P3	II, III, IVa, MB
	alaat_1P5	III, IVa, MB
Aspartate aminotransferase (ASP-AT)	*aspat_1P1*	II, III, MB
	aspat_1P2	IVa
	aspat_2P3	Ang, MB
	aspat_3P4	Ang, III, IVa, MB
Carbonic anhydrase (CA)	*ca_1P1*	MB
	ca_2P2	III
	ca_2P3	Cim, II, III, IVa, IVb, MB
Dicarboxylate carrier (DIC)	*dic_1P1*	Cim, II, III, IVa, MB
Dicarboxylate transporter (DIT)	-	no duplications
Glyceraldehyde-3-phosphate dehydrogenase (GAPDH)	*gapdh_2P1*	II, III, IVa, IVb, MB
	gapdh_2P2	IVa
	gapdh_3P1	Ang
NAD-dependent malate dehydrogenase (NAD-MDH)	*nadmdh_1P1*	II, IVb
	nadmdh_1P8	Cim
	nadmdh_2P4	III
	nadmdh_3P5	II, III, IVa, IVb, MB
NAD-malic enzyme (NAD-ME)	*nadme_1P1*	Cim, IVa
	nadme_2P2	IVa
NADP-dependent malate dehydrogenase (NADP-MDH)	*nadpmdh_1P1*	IVa, MB
NADP-malic enzyme (NADP-ME)	*nadpme_1P1*	Cim, III, IVa, IVb, MB
	nadpme_1P2	Ang, IVa
	nadpme_1P4	Cim, Ang, III, IVa, MB
PEP carboxykinase (PCK)	*pck_1P1_LGT:C*	Ang, III, IVa, IVb, MB
PEP carboxylase kinase (PEPC-K)	*pepck_1P3*	Ang
	pepck_3P6	IVa
Pyruvate kinase (PK)	*pk_1P1*	Cim, Ang, II, III, IVa, IVb, MB
	pk_1P2	MB
Inorganic pyrophosphatase (PPA)	*ppa_2P1*	IVa, MB
	ppa_3P1	MB
	ppa_4P1.6	III
PEP carboxylase (PEPC)	*ppc_1P3*	Ang, II, III, IVa, IVb, MB
	ppc_1P3_LGT:A	IVb
	ppc_1P3_LGT:M	Cim, III
	ppc_1P6	Cim, IVb

	ppc_1P7	II
Phosphoglycerate kinase (PGK)	-	no duplications
Pyruvate phosphate dikinase (PPDK)	*ppdk_1P1*	I, II, IVb, MB
	ppdk_1P2	MB
Pyruvate phosphate dikinase regulatory protein (PPDK-RP)	-	no duplications
PEP-phosphate translocator (PPT)	*ppt_1P4*	Ang, III, IVa
	ppt_1P6	II, III, IVa, IVb, MB
Sodium bile acid symporter 1 (SBAS)	-	no duplications
Tonoplast malate/fumarate transporter (TDT)	*tdt_1P2*	Ang, I, IVa, IVb, MB
Triosephosphate-phosphate translocator (TPT)	*tpt_1P2*	Cim

[1] I, II, III and IV refers to the nuclear clades of *A. semialata* (Olofsson *et al.*, 2016); Ang is *A. angusta*; Cim is *A. cimicina*; MB refers to *A. semialata* individuals with mixed genetic background (Olofsson *et al.*, 2016).

Table S4. Allele-specific expression analyses.

Gene	Accession	Type	Sequencing depth	Number of SNPs	Mean depth[1] (transcript)	Mean depth[1] (genome)	Regression analysis[2]		
							Slope	R^2	*p*-value
pck_1P1_LGT	AUS1	C_4	low-coverage	5	12,577	4.2	0.27	0	0.46
pck_1P1_LGT	BUR1	C_4	low-coverage	6	9,038	5	**1.6**	**0.91**	**< 0.001**
pck_1P1_LGT	MAD1	C_4	low-coverage	5	595	5	**0.84**	**0.93**	**0.01**
pck_1P1_LGT	TAN4	C_4	low-coverage	6	1,145	4	0.08	0	0.38
pck_1P1_LGT	TPE1-3	C_4	low-coverage	4	2,372	4.2	-0.19	0	0.57
pck_1P1	BUR1	C_4	low-coverage	5	284	1.4	0.06	0	0.71
pck_1P1	TAN2-A	C_3+C_4	low-coverage	4	70	1.2	-	-	-
pck_1P1	TAN4	C_4	low-coverage	4	38	1.5	0.03	0.41	0.22
ppc_1P3_LGT_A	AUS1	C_4	low-coverage	4	3,631	2.5	0.28	0.7	0.1
ppc_1P3_LGT_M	BUR1	C_4	low-coverage	5	6,724	2.2	-0.13	0	0.81
ppc_1P3_LGT_M	MAD1	C_4	low-coverage	4	181	2.8	0.12	0	0.45
ppc_1P3	BUR1	C_4	low-coverage	77	7,760	12.2	0.2	0.07	0.01
ppc_1P3	MAD1	C_4	low-coverage	58	748	9.3	0.03	0	0.74
ppc_1P3	RSA2	C_3	low-coverage	46	268	2.8	0.03	0.05	0.09
ppc_1P3	TAN1	C_3+C_4	low-coverage	4	859	8.8	0.06	0	0.74
ppc_1P3	TAN2-A	C_3+C_4	low-coverage	4	1,330	3.8	0.26	0.74	0.09
ppc_1P3	TAN4	C_4	low-coverage	22	50	6.4	0.17	0.13	0.06
ppc_1P3	TPE1-3	C_4	low-coverage	8	2,572	6.5	-0.06	0	0.38
ppc_1P6	TPE1-3	C_4	low-coverage	30	20,032	8.4	**0.21**	**0.23**	**< 0.001**
pck_1P1_LGT	TPE1-10	C_4	high-coverage	4	2,372	158.8	1.26	0.75	0.09
pck_1P1	TAN2-A	C_3+C_4	high-coverage	4	70	5.5	-0.04	0	0.8
ppc_1P3	TAN2-A	C_3+C_4	high-coverage	4	1,330	20	0.48	0.38	0.23
ppc_1P3	TPE1-10	C_4	high-coverage	8	2,572	181.9	0.61	0.38	0.06
ppc_1P6	TPE1-10	C_4	high-coverage	30	20,032	209.3	**0.99**	**0.93**	**< 0.001**

[1] Mean number of reads covering each SNP; [2] Linear regression of the depth of the minor allele in the transcriptome and genome datasets.

Table S5. Effect of phylogenetic tree on the phylogenetic generalized least squares (PGLS) analysis used to test for an association between changes in gene copy number and changes in transcript abundance.

Gene family	*p*-value range[1]
Alanine aminotransferase (ALA-AT)	0.041 – 0.279
Aspartate aminotransferase (ASP-AT)	0.288 – 0.536
Carbonic anhydrase (CA)	0.392 – 0.62
Dicarboxylate transporter (DIT)	-
NAD-malate dehydrogenase (NAD-MDH)	0.061 – 0.224
NAD-malic enzyme (NAD-ME)	0.499 – 0.633
NADP-malate dehydrogenase (NADP-MDH)	-
NADP-malic enzyme (NADP-ME)	0.405 – 0.591
PEP carboxykinase (PCK)	0.001 – 0.006
PEP carboxylase (PEPC)	< 0.001
Pyruvate phosphate dikinase (PPDK)	0.798 – 0.835
PEP-phosphate translocator (PPT)	0.557 – 0.764
Sodium bile acid symporter (SBAS)	-
Triosephosphate-phosphate translocator (TPT)	-

[1] *p*-value ranges are the interquartile range of PGLS fitting computed using 100 independent Bayesian trees. Before the analysis, transcript abundance values were log10 transformed and copy numbers were expressed as integers. Gene families lacking *p*-values do not show copy number variation, or contain representatives with no gene sequence available for the phylogenetic analysis. *p*-value ranges in bold include statistically significant results after correcting the significance level ($\alpha = 0.05$) for multiple comparisons.
